# Supplementary material for: Heatwaves, hospitals and health system resilience in England: a qualitative assessment of frontline perspectives from the hot summer of 2019
Source: BMJ Open. 2023 Mar 6;13(3):e068298. doi: 10.1136/bmjopen-2022-068298 (PMC9990610; doi:10.1136/bmjopen-2022-068298)
Supplement: Author's manuscript [file bmjopen-2022-068298.draft_revisions.pdf]

BMJ Open is committed to open peer review. As part of this commitment we make the peer review history of every article we publish publicly available.

When an article is published we post the peer reviewers' comments and the authors' responses online. We also post the versions of the paper that were used during peer review. These are the versions that the peer review comments apply to.

The versions of the paper that follow are the versions that were submitted during the peer review process. They are not the versions of record or the final published versions. They should not be cited or distributed as the published version of this manuscript.

BMJ Open is an open access journal and the full, final, typeset and author-corrected version of record of the manuscript is available on our site with no access controls, subscription charges or pay-per-view fees (http://bmjopen.bmj.com).

If you have any questions on BMJ Open's open peer review process please email info.bmjopen@bmj.com

BMJ Open

Heatwaves, hospitals, and health system resilience in England: a qualitative assessment of frontline perspectives from the hot summer of 2019

Journal:	*BMJ Open*
Manuscript ID	bmjopen-2022-068298
Article Type:	Original research
Date Submitted by the Author:	13-Sep-2022

Complete List of Authors:	Brooks, Katya; UK Health Security Agency (UKHSA), Extreme Events and Health Protection team<br>Landeg, Owen; UK Health Security Agency (UKHSA), Extreme Events and Health Protection team<br>Kovats, Sari; London School of Hygiene & Tropical Medicine, Dept of Social and Environmental Health Research<br>Sewell, Mark; NHS Improvement, National Emergency Preparedness Resilience and Response<br>OConnell, Emer; UK Health Security Agency, Extreme Events and Health Protection team

Keywords:	PUBLIC HEALTH, Health & safety < HEALTH SERVICES ADMINISTRATION & MANAGEMENT, Quality in health care < HEALTH SERVICES ADMINISTRATION & MANAGEMENT, INTENSIVE & CRITICAL CARE, OCCUPATIONAL & INDUSTRIAL MEDICINE, QUALITATIVE RESEARCH

SCHOLARONE™
Manuscripts

I, the Submitting Author has the right to grant and does grant on behalf of all authors of the Work (as defined in the below author licence), an exclusive licence and/or a non-exclusive licence for contributions from authors who are: i) UK Crown employees; ii) where BMJ has agreed a CC-BY licence shall apply, and/or iii) in accordance with the terms applicable for US Federal Government officers or employees acting as part of their official duties; on a worldwide, perpetual, irrevocable, royalty-free basis to BMJ Publishing Group Ltd ("BMJ") its licensees and where the relevant Journal is co-owned by BMJ to the co-owners of the Journal, to publish the Work in this journal and any other BMJ products and to exploit all rights, as set out in our licence.

The Submitting Author accepts and understands that any supply made under these terms is made by BMJ to the Submitting Author unless you are acting as an employee on behalf of your employer or a postgraduate student of an affiliated institution which is paying any applicable article publishing charge ("APC") for Open Access articles. Where the Submitting Author wishes to make the Work available on an Open Access basis (and intends to pay the relevant APC), the terms of reuse of such Open Access shall be governed by a Creative Commons licence – details of these licences and which Creative Commons licence will apply to this Work are set out in our licence referred to above.

Other than as permitted in any relevant BMJ Author's Self Archiving Policies, I confirm this Work has not been accepted for publication elsewhere, is not being considered for publication elsewhere and does not duplicate material already published. I confirm all authors consent to publication of this Work and authorise the granting of this licence.

For peer review only

Title: Heatwaves, hospitals, and health system resilience in England: a qualitative assessment of frontline perspectives from the hot summer of 2019

Authors: Katya Brooks[1], Owen Landeg[1], Sari Kovats[2] (corresponding author), Mark Sewell[3], Emer OConnell[1]

[1]Extreme Events and Health Protection, UK Health Security Agency (UKHSA); katya

[2] NIHR Health Protection Research Unit in Environmental Change and Health, Faculty of Public Health and Policy, London School of Hygiene and Tropical Medicine

[3]NHS England and NHS Improvement

Conflicts of interest: None identified

Abstract: [228 words]

Objective: To critically assess the impacts of very hot weather on i) frontline staff in hospitals in England; and ii) on health care delivery and patient safety.

Study design: Qualitative assessment using key informant interviews and thematic analysis.

Setting: England

Participants: 14 employees of the NHS (clinicians, non-clinicians including facilities managers and emergency preparedness, resilience and response (EPRR) professionals).

Results: Hot weather in 2019 caused significant disruption to health services, facilities and equipment, staff and patient discomfort, and an acute increase in hospital admissions. Levels of awareness varied between clinical and non-clinical staff of the Heatwave Plan for England, Heat-Health Alerts and associated guidance. Responding to heatwaves was also

For peer review only - http://bmjopen.bmj.com/site/about/guidelines.xhtml

affected by competing priorities and tensions (including infection control, electric fan usage and patient safety).

Conclusions: Healthcare delivery staff experience difficulty in managing heat risks in hospitals. Priority should be given to workforce development and strategic, long-term planning, prevention and investment to enable staff to prepare and respond, as well as to improve health system resilience to current and future heat-health risks. Further research with a wider, larger cohort is required to develop the evidence base on the impacts, including the costs of those impacts, and to assess the effectiveness and feasibility of interventions. Forming a national picture of health system resilience to heatwaves will support national adaptation planning for health, in addition to informing strategic prevention and effective emergency response.

Key words: hot weather, high temperatures, climate change, adaptation, public health, emergency planning, patient safety

Strengths and limitations

Although a small number of healthcare staff was interviewed, this included both clinical and non-clinical staff providing different perspectives; however, relatively fewer clinical staff agreed to be interviewed so the study lacks insight from across different aspects of clinical care (emergency, palliative, etc). Interviews were conducted several months after the event, thereby possibly introducing recall, recruitment and interviewer bias.

1. Introduction

For peer review only - http://bmjopen.bmj.com/site/about/guidelines.xhtml

The frequency, intensity and duration of heatwaves is increasing in the United Kingdom (UK) due anthropogenic climate change.(1, 2, 3) Heatwaves are already a public health concern and are considered a risk to national security.(4) The UK's Third Climate Change Risk Assessment (CCRA3) concluded that risks to population health, health system resilience and clinical care from increased exposure to heat in homes and other buildings is a priority risk for urgent action for England.(5) Summer 2019 was warmer than average; the highest daily temperature recorded to date in England (38.7°C) was recorded in Cambridge on 25th July.(6) A total of 15 Level 2 or Level 3 Heat-Health Alerts were issued across all regions of England in June, July and August 2019 based on the thresholds for the Heatwave Plan for England. (7)

The Heatwave Plan (7) aims to raise awareness of the health consequences of hot weather and describes what health care professionals can do to prepare and plan for hot weather along with actions to take in response to a heatwave. Actions for frontline staff in primary and secondary care include: identify and check on high-risk individuals and raise awareness of heat illnesses and their prevention among clients and carers; identify or create cool rooms/areas where temperatures can be maintained below 26°C and regularly record indoor temperatures and; ensure business continuity plans are in place and implemented as required. (7)

An estimated 90% of hospital buildings are vulnerable to overheating (8) and NHS estates are at risk of high indoor temperatures (overheating) even during moderately warm summers (9); temperatures in some wards can exceed 30˚C even when external temperatures are 22˚C.(10) Existing standards for healthcare premises recommend temperatures from 18˚C to 28˚C in general wards and 18˚C to 25˚C for more sensitive areas,

For peer review only - http://bmjopen.bmj.com/site/about/guidelines.xhtml

such as birthing and recovery rooms.(11) In 2019-20, there were 3,600 instances of overheating above 26°C reported in NHS Trust buildings in England.(12) In addition to concerns about patient safety, heat is an occupational health hazard and can cause discomfort and harm to staff as well as affect productivity.(13, 14, 15)

Limited observational evidence currently exists about how healthcare settings manage episode of hot weather. Anecdotal evidence for impacts has been reported during heatwave events.(16, 17, 18). This study aims to explore the experiences of staff during hot weather in healthcare facilities in England in summer 2019 with a focus on the impacts on health service delivery, staff welfare, and patient safety.

2. Methods

Qualitative insight data were collected using a pre-interview survey and semi-structured interviews. Ethical approval was granted by PHE's Research Ethics and Governance Group in September 2019 (R&D reference: 366).

Participants were identified using snowball, purposive sampling to include those with a broad range of clinical and non-clinical expertise from healthcare facilities across England. Sample size was determined by applying the concept of saturation, with suggested individuals being invited and interviewed until no new themes or issues emerged, within the scope of available resources. Participants were invited by email to a one-hour telephone/Skype or face-to-face interview, depending on their geographical location, availability and preference. On acceptance, they were invited to complete a short pre-interview online questionnaire in SelectSurvey, covering actions related to each Heat-Health Alert level (Level 2 and Level 3) from the Heatwave Plan to inform discussions during the interview. All participants provided written consent prior to participation.

For peer review only - http://bmjopen.bmj.com/site/about/guidelines.xhtml

Semi-structured interviews provided an open framework to engage with participants on their experiences, views and reflections of working in healthcare estates during summer 2019. An interview protocol and topic guide, based around a schedule of common questions, was piloted and employed to ensure consistency and enable comparison across interviews. For accuracy and transparency, interviews were recorded and transcribed verbatim, and all participants given the opportunity to review their interview transcripts. The dataset was thematically analysed and coded in EPPI-Reviewer using a coding framework constructed through an iterative process that was both inductive and deductive, and trialled and agreed by the research team.

3. Results

Fourteen semi-structured interviews were completed by key informants in hospital settings comprising four clinical and 10 non-clinical employees of the NHS (including Estates and Facilities and Emergency Preparedness, Resilience and Response (EPRR) professionals) (Table 1) between October 2019 and January 2020.

Table 1. Breakdown of interviews by interview and role type

Code	Role type	Region
Non-clinical 1	Non-clinical (EPRR)	London
Non-clinical 2	Non-clinical (critical care)	London
Clinician 1	Clinician (acute medicine)	West Yorkshire
Non-clinical 3	Non-clinical (NHS E&I)	National / London

For peer review only - http://bmjopen.bmj.com/site/about/guidelines.xhtml

Code	Role type	Region
Non-clinical 4	Non-clinical (EPRR)	London
Clinician 2	Clinician (acute medicine)	Liverpool
Clinician 3	Clinician (junior doctor)	London
Non-clinical 5	Non-clinical (EPRR)	London
Non-clinical 6	Non-clinical (EPRR)	Berkshire
Non-clinical 7	Non-clinical (EPRR)	Kent
Non-clinical 8	Non-clinical (estates and facilities)	London
Clinician 4	Clinician	Manchester
Non-clinical 9	Non-clinical (EPRR)	London
Non-clinical 10 and Non-clinical 11 (Interviewed together)	Non-clinical (EPRR)	Kent

DRAFT

For peer review only

Thematic analysis identified the following key themes: impacts of heat on individual health and wellbeing (occupational health, and patient health and safety); impacts on healthcare provision; barriers to service delivery heatwave planning and consideration of adaptation to climate change.

Impacts of heat on staff and patients

The majority of participants reported experiencing overheating in healthcare buildings during the summer, with adverse effects on themselves and other staff and patients. Clinical and non-clinical respondents reported significant impacts of high temperatures on both staff and patients in terms of feeling uncomfortable, tired, stressed, unable to cope, less efficient, and observing distress in patients.

> *'It had a huge impact on staff, but also on the patients as well, especially on the wards… some of them were acutely unwell at that time and exacerbated by very uncomfortable conditions'* (Non-clinical 14).

> *I remember… running around trying to get fans for the patients but the nurses…not having anything at the nurses' station, and people were generally just hot and stressed and looked uncomfortable. I remember one time we abandoned the handover because we just didn't cope in that room.* (Clinician 2)

Some facilities may be particularly prone to overheating due to factors in addition to building design. One EPRR Manager for a psychiatric unit reported that:

> *Especially for those patients… who are detained under the Mental Health Act and aren't allowed, or it's not safe for them to leave any of the wards…. some of the windows can't be opened for obvious reasons…. I know there was a few issues, understandably, family members were getting extremely distressed with staff saying, you know, this is unacceptable, [and I] totally understand it. … you have some kind of frustrations there amongst patients. We could see a rise in disruptive behaviour on the wards."* (Non-clinical 9)

For peer review only - http://bmjopen.bmj.com/site/about/guidelines.xhtml

A high level of goodwill for maintaining patient care in the face of challenging working conditions on the wards was recognised by frontline clinicians: '*People did kind of pull together in adversity and everyone tried to keep the whole thing working'* (Clinician 3); *'That's probably the motto of the NHS: "just get on with it"'* (Clinician 4), as well as non-clinical staff: *'Our staff are really adaptable: whether it's hot or cold, they're really adaptable and the patients are at the heart of whatever they do and they check if our patients are ok in their own homes. Our staff are fantastic, they are brilliant'* (Non-clinical 10/11).

Acute increases in health service use, particularly for vulnerable adults

Respondents reported an increase in admissions on hot days: *'…you definitely notice that it's busier'* (Clinician 2). Patients were reported to present with physical symptoms of heat exhaustion, dehydration, cardiovascular and respiratory conditions, as well as '*injuries related to people doing kind of [outdoor] activities that you do in hot weather; so cycling, going on motorbike riding, drinking alcohol, things like that….'* (Clinician 4).

A specialist in acute medicine, reflected: *' [As well as] elderly people getting dehydrated, getting low blood pressure and having to come in because of fainting episodes and things like that and vulnerable groups that aren't able to rehydrate themselves, ask for water or reach the water because they've got dementia, or are very frail….'* (Clinician 4).

In addition to clinical risk factors, clinical respondents reported patients' housing or socio-economic status as putting them at greater risk in hot weather: *'[With one patient I remember] it was like late evening, so you can't speak to the GP and I remember kind of thinking, "My god, we're going to send him back to his [hot] flat which sounds like it's really precipitating continuous seizures and I don't know what kind of longer-term effects that's having…."'* (Clinician 3).

For peer review only - http://bmjopen.bmj.com/site/about/guidelines.xhtml

There were also concerns about discharging patients. Only two respondents, both EPRR Managers, reported protective procedures in place:

> *…[for] patients who are being discharged home during a Level 3 heatwave… we have a discharge proforma that gets filled in…. It highlights…these people will be at risk if they're on their own in a top floor flat with drugs that inhibit sweating, or inability to be walking around and getting drinks and keeping cool, for example. And then we would give those people a copy of the PHE [Beat the Heat] leaflet. Or is it safe to actually get them at home with some kind of social services input?* (Non-clinical 7)

Social isolation was also raised as a compounding factor: '*… people who were more socially isolated, perhaps living on their own, just managing most of the time, then weren't managing'* (Clinician 1).

Impacts of heat on facilities and equipment

Significant impacts on medical equipment and facilities that led to impacts on healthcare provision were reported. In relation to medical equipment, an EPRR Manager experienced failure of medication storage facilities in a psychiatric unit: *'Some of our medication, especially the anti-psychotic medication, needs to be kept at a specific temperature…. I believe on a couple of occasions the actual fridge which was containing some medication had bizarrely overheated, or just broke down'* (Non-clinical 9). Two respondents reported ICT issues due to server rooms overheating and as a result: *'they lost quite a lot of IT aspects, including the ability for all their different systems to talk to each other'* (Non-clinical 3).

An Estates and Facilities Manager reported high indoor temperatures in critical care units, operating theatres and rooms with MRI scanners which caused disruptions in patient care,

For peer review only - http://bmjopen.bmj.com/site/about/guidelines.xhtml

from loss of beds (which became unusable) and loss of equipment (scanners became unusable).

> *The intensive care unit was probably our worst area, and we struggled with some of our [MRI] scanners to keep them cool in certain periods as well, and that would have impacted on patient care. The paediatric scanning was suspended for periods; it was too unpleasant for the young ones to go through it.* (Non-clinical 8)

This view was echoed by an EPRR Manager from the same hospital:

> *Probably because the heat was rising and it just put enormous pressure on [our air conditioning unit] but the contingency didn't work, which meant that ... half of the ITU beds were lost or they were overheating…. We had to look at evacuating the critical care area because it was just getting too hot…. We moved some vulnerable out of those areas…. There were theatre suites that ... because the air conditioning was working so hard, they got full condensation so there was literally water running down on the walls and they had to stop operating… just to dry the walls, then deep clean it and then continue with the theatre list.* (Non-clinical 5)

Critical care facilities have a large number of air conditioners. These two respondents also reported that the increased demand during the hot weather caused a power failure which exacerbated indoor temperatures due to lack of air conditioning and also caused problems for refrigeration services as well as the mortuary: '*…. the mortuary fridges failed. In response, the Estates and Facilities Team 'converted a container for a temporary mortuary'* (Non-clinical 8). As a direct consequence of these concurrent events and their cumulative impacts on service delivery and patient safety, this hospital declared an internal incident due to the heatwave.

DRAFT
For peer review only

For peer review only - http://bmjopen.bmj.com/site/about/guidelines.xhtml

Barriers to service delivery in a heatwave

Several barriers to delivery of care in a heatwave were identified by respondents. These have been categorised in terms of preparedness and response to Heat-Health Alerts, built environment and managing indoor temperatures, and behaviours.

Preparedness and response to Heat-Health Alerts

Lack of time and capacity to respond to high temperatures due to competing priorities for frontline staff were reported across clinical and non-clinical respondents:

> *…Often when these things happen you're just so, so busy and it sometimes feels like you go from one crisis to another, that you don't actually have an awful lot of time to step back and say: "Right. What can we do differently?"* (Clinician 2)

Data from pre-interview questionnaires indicate a greater lack of awareness of the Heatwave Plan for England and Trust-level plans and Heat-Health Alerts amongst clinical respondents as compared with non-clinical respondents (see Figure 1).

Figure 1. Pre-interview questionnaire responses: awareness of the Heatwave Plan for England and the Heat-Health Alerts system

INSERT FIGURE

The majority of respondents reported that they received or cascaded Heat-Health Alerts by email. However, no clinician could recall receiving the alerts: '*Realistically as a [Junior Doctor] you're probably not logging on to your emails, your NHS emails, that often*' (Clinician 3). A reality recognised by some EPRR practitioners: '*[Email is] the best way to communicate*

For peer review only - http://bmjopen.bmj.com/site/about/guidelines.xhtml

really on heatwave alerts…. But, will they look at it? No way, not really' (Non-clinical 5) and *'It's a kind of, they'll see on the news or something'* (Non-clinical 9).

Across clinical respondents, a significant difference was observed in levels of awareness of the heat-health risks in general, in comparison with winter and cold weather:

> *Hot weather is never something that I've heard mentioned. Hot weather is almost one of those things you just get on and deal with….. Whereas all the time, even in the middle of summer, we're going to a wrap-up last winter meeting and then you go to a, what are we going to do next winter meeting.* (Clinician 2)

The majority of EPRR professionals interviewed identified the need for strategic, long-term planning, preparation and investment across the system to ensure healthcare resilience to all extreme weather hazards: *'The key thing for me, is about relationship building with [Estates and Facilities], so we will work closely with them all year round, not just when we suddenly get a heatwave alert'* (Non-clinical 7).

> *We need to be much more prepared…. it's kind of frustrating really that we kind of deal with it when we're in the midst of it, you know…. So I think the lessons that we've learnt is almost we need to prepare from the turn of the year.* (Non-clinical 9)

> *Sometimes it's a challenge to get people to start thinking long term, and investment, money will always come down to being part of the issue….* (Non-clinical 1)

> *[The Heatwave Plan] gets circulated [annually] before our heat season starts and for people to say, please remember this. But the reality is that the majority of people have never opened it up and they'll kind of get almost kind of hit by it on the day*

For peer review only - http://bmjopen.bmj.com/site/about/guidelines.xhtml

when it gets hot. It's unfortunate it's a very reactive plan, rather than concentrating on this year-round. (Non-clinical 5)

Challenges of managing indoor temperatures

Challenges of managing indoor temperatures often arise from the design of NHS buildings, which vary in the insulation and ventilation characteristics. Indoor temperatures are also increased by indoor heat sources (computers and equipment). Adaptation measures are limited by a lack of financial investment in the management of healthcare estates. One EPRR Manager reflected: *'The NHS is ageing and there's very little financially that we can do about it….'* (Non-clinical 5). A financial barrier reported by three non-clinical respondents stems from outsourced ownership of healthcare estate buildings, property management and maintenance leading to lack of control in making decisions to manage internal temperatures:

> *The majority of buildings are not owned by us, they are owned by NHS property services and we are tenants, and that's where we tend to get problems: There's normally quite a lot that's out of our control as it's a property services building so we can't necessarily do what we'd ideally want to do…. It would be nice to take control of own destinies.* (Non-clinical 10/11)

There is some evidence that new buildings are more adapted to hot weather. An EPRR Manager responsible for two sites commented that: *… Our [new] site, which is designed to kind of withstand heatwaves, we didn't have to activate any contingencies there at all… whereas at [the older site]… we definitely see that increase in [internal] temperature here…. we were getting… multiple incident forms every day in the summer.* (Non-clinical 7)

For peer review only - http://bmjopen.bmj.com/site/about/guidelines.xhtml

Infection prevention and control policy was identified as a significant barrier to the use of electric fans, and to a lesser extent, air conditioning units in ward settings by all clinical and the majority of non-clinical respondents: '*Infection control is one of the overriding mantras everywhere'* (Clinician 1). Six respondents reported complete bans on electric fan usage in clinical areas 'W*e were just told that due to infection control we weren't allowed to use [electric fans] for patients'* (Clinician 4); '*They wouldn't allow fans because they said it was a health and safety risk'* (Clinician 3); '*…a clinical alert came out regarding…the fans…and said that… they're not to be used in clinical areas…. Because of infection control…and that came out just before the heatwave, you know, which made it quite difficult*' (Non-clinical 9). Others reported fans being used as expected, with inpatient areas prioritised: '*I remember people…running around trying to get fans for the patients and the nurses sort of not having anything at the nurses' station*' (Clinician 2). The difficulty of ensuring enough fans were available to provide sufficient cooling during heatwaves was cited: '*We had fans…. So all that was kind of in place, there just wasn't enough of it…. It's impossible to test like how many fans does it take to cool down this ward when it gets to 34 degrees*' (Non-clinical 5).

Two EPRR Managers stated that, due to financial and infection control policy constraints, the decision was made in their hospitals to dispose of electric fans and buy replacements annually to benefit patient care, if not the environment: '*Is it better to cool down the infectious patients with the fan, you know, and then chuck it, the fan, afterwards if you can't clean it, or let the poor man or woman just melt?'* (Non-clinical 5) and, *'This is not great for the environment but it's cheaper actually just to bin them and buy new fans'* (Non-clinical 1).

For peer review only - http://bmjopen.bmj.com/site/about/guidelines.xhtml

Most EPRR respondents reported annual procurement of portable air conditioning units: ‘*Where it's possible we bring in mobile air con units, but that's obviously not an ideal response’* (Non-clinical 4).

> *We can't purchase something portable and keep them in a cupboard, it's not a cost-effective way to do this. We will have to use an external company who can store them; you know, maintain them and test them and will bring them on site and hire them for that period of time.* (Non-clinical 5)

Two respondents reported instances where air conditioning could not be used across their sites: ‘*A lot of our corporate settings had the portable air conditioning units that could be used, but of course they can't be used in clinical settings because of infection control*’ (Non-clinical 9).

> *There were five…inpatient areas in old buildings that didn't have air conditioning installed, couldn’t - either for the size or the location of the ward, or the complexity of the patients’ [needs]…. We had outpatient areas where there could be children; you can't put any kind of air conditioning unit because of the risk of kiddies touching them. The only place with air conditioning in some areas would be in the middle of the nurses' station because of the risk of trip hazards, of the wires and of course, to elderly patients.* (Non-clinical 5)

Participants called for increased clarity of infection control guidance at a national level as ‘*different trusts’ infection control people say different things’* (Non-clinical 7) in order ‘*to demystify everything. For example, that air conditioning can or can’t be used due to legionella’* (Non-clinical 6).

DRAFT

For peer review only

Participants questioned the feasibility of actions outlined in the Heatwave Plan, to create cool rooms and for checking indoor temperatures.

> *…Every bed is almost always full and there isn't necessarily extra space in the hospital to have a cooler area or the infrastructure to cool an area down…. it would be very challenging to implement actually, and I think… it would be difficult to kind of move someone out of the cool area into the hotter area in exchange for another patient, because you're subjecting them to a bit more discomfort and possibly risk as well.* (Clinician 4)

> *… What if your coolest room is not near your nursing station, but you're going to put your vulnerable patients in there? ... Is it right that we put a vulnerable patient further away just because it's a bit cooler than anywhere else?* (Non-clinical 1)

<u>The role of adaptive behaviours</u>

Heat risks can be reduced by changes in behaviour, in both frontline staff and patients. All participants reported lack of knowledge as a major challenge to adopting cooling strategies in health facilities, such as closing curtains or blinds, moving patients, etc.: *'…There doesn't appear to be any plan on it, but I think it was down to… nurses thinking we need to keep this patient in the shade'* (Clinician 1).

Workplace culture and attitudes were raised in relation as a barrier to staff drinking water and keeping hydrated whilst working on the ward due to fear of appearing unprofessional. One EPRR Manager sought to address this problem: '*We made it very clear to staff that we wanted to see them carrying water around, we wanted to see them drinking on duty, just to dispel all this old nonsense about, "oh you shouldn't be seen drinking on duty, it's*

DRAFT
For peer review only

For peer review only - http://bmjopen.bmj.com/site/about/guidelines.xhtml

unprofessional". Actually, we said: "It is professional, it's looking after yourself"' (Non-clinical 7).

4. Discussion

This study has shown the heatwaves can cause significant impacts on staff and patient wellbeing as well as health service delivery. Many NHS estates are not sufficiently adapted to hot weather and this will become more disruptive as the hot weather increases year on year due to climate change unless adaptation measures are taken. This research highlights the current level of preparedness and capacity to respond to current heat episodes. Formal debriefs of overheating events are rarely undertaken by hospitals. This work will inform the UK Health Security Agency's commitment to develop a single adverse weather and health plan under the second National Adaptation Programme (2018-2023).

Our findings demonstrate the wide range of impacts in health care settings, including increased demand, discomfort of patients and staff, and rooms and equipment failing or becoming unusable leading to disruptions in service. Studies of individual hospitals and wards have indicated the factors that lead to overheating (for example, 16, 18) but it is also important to understand how staff behaviour and clinical practice change during heat episodes and how this may also affect patient care.

The findings highlight barriers to care delivery in a heatwave associated with preparedness and response, the built environment and managing indoor temperatures, and behaviours. Insufficient capacity, difficulties in prioritisation of risk mitigating actions, gaps in awareness of heat-health risks and understanding of actions to take on receipt of an alert were all cited. These findings correlate with those of the *Evaluation of the Heatwave Plan for England*'s 2019 national survey of nurses in hospital, community and care home settings

For peer review only
DRAFT

For peer review only - http://bmjopen.bmj.com/site/about/guidelines.xhtml

which reported that “many frontline staff, including nurses surveyed, reported to be unaware of any local heatwave plans, and unfamiliar with the Heatwave Plan guidance…many reported taking few or none of the recommended Heatwave Plan actions during an alert”.(19) This study also reported issues with service delivery due to lack of organisational preparation for extreme heat events, a lack of funding and resources, as well as working in challenging conditions in buildings not well adapted for a changing climate.

Our findings highlight the need for improvements in how Heat-Health Alerts and associated advice are communicated, particularly to frontline staff across the wider health system. Efforts on workforce development should focus on empowering staff to understand and act upon Heat-Health Alerts and to embed adaptive behaviours in their daily practice. Ensuring a climate resilient health system will require strategic, long-term planning and investment to address the drivers for overheating risk and the barriers to action both before and during a heatwave event.

Overheating in hospital and healthcare facilities remains a serious and escalating problem. The UK Government’s Health Infrastructure Plan includes an aim to build 48 new hospitals by 2030; to avoid unintended harms, it is essential that the goals of climate adaptation and mitigation are considered in parallel. Actions to improve energy efficiency and building design to achieve NHS Net Zero targets must also consider the risk of overheating.

This research provides important insights on operational barriers to action, particularly in relation to use of electric fans as a cooling strategy and IPC concerns. Implementation of individual cooling interventions should form part of a whole systems approach that considers a hierarchy of cooling interventions, with active cooling as a last resort. The approach to cooling for any indoor environment should take account of the effectiveness,

For peer review only - http://bmjopen.bmj.com/site/about/guidelines.xhtml

374 cost, feasibility, ease of use and sustainability of the different options in that context – in
375 some settings, a combination of options may work best. This study has highlighted that a
376 lack of clarity or consistency of policy on the use of fans and/or cooling systems has resulted
377 in heat mitigations not being used and/or costly work-arounds being put in place due to
378 concerns around IPC. Heat is one of several risks and inevitably trade-offs must be made.
379 Further, the routine disposal and replacement of electric fans does not align with Net Zero
380 commitments.

381 This study has a number of limitations. Interviews were conducted several months after the
382 event, thereby possibly introducing recall, recruitment and interviewer bias. The relatively
383 low engagement from clinicians in comparison with non-clinical NHS representatives may
384 introduce a bias in terms of perspective.

385 **5. Conclusion**

386 This study confirms for the first time that heatwaves can cause significant disruption to
387 health service delivery in England. National public health systems are developing methods,
388 plans and tools to manage climate risks, but adaptation is still largely viewed as an
389 emergency response activity. Further action is needed to improve the climate resilience of
390 the health system, in particular to address overheating in hospitals and residential care
391 buildings. (20) Additional research is recommended to further develop the evidence base on
392 the impacts, viability of interventions and current costs associated with heat-related
393 disruption to health service delivery. This should inform a broader assessment of the climate
394 vulnerability of the health system, and future planning assumptions for strategic prevention
395 and emergency response.

For peer review only - http://bmjopen.bmj.com/site/about/guidelines.xhtml

6. Acknowledgements

The authors wish to thank the stakeholders that participated in this research.

7. Author statement

Contributors: KB and OL contributed equally to this paper. KB and OL designed the study, collected and analysed the data, drafted and revised the paper. MS contributed to the design and implementation of the study. SK contributed to the design of the study. EO contributed to the design of the study, critically reviewed and finalised the draft paper.

8. Funding statement

This work was supported by the National Institute for Health Research (NIHR) Health Protection Research Unit in Environmental Change (NIHR200909), a partnership between the UK Health Security Agency (UKHSA) and the London School of Hygiene & Tropical Medicine. The views expressed are those of the author(s) and not necessarily those of the NIHR, UKHSA or the Department of Health and Social Care.

References

1. International Panel for Climate Change (2021): Summary for Policymakers. In: Climate Change 2021: The Physical Science Basis. Contribution of Working Group I to the Sixth Assessment Report of the Intergovernmental Panel on Climate Change [Masson-Delmotte, V., P. Zhai, A. Pirani, S.L. Connors, C. Péan, S. Berger, et al.]. Cambridge University Press, Cambridge, United Kingdom and New York, NY, USA, pp. 3−32, doi:10.1017/9781009157896.001

For peer review only - http://bmjopen.bmj.com/site/about/guidelines.xhtml

2. Mitchell D, Heaviside C, Vardoulakis S, Huntingford C, Masato G, Guillod B et al. (2016) Attributing human mortality during extreme heatwaves to anthropogenic climate change. *Environ. Res. Lett.* 11(7):8. 074006

3. Carmichael C, Bickler G, Kovats S, Pencheon D, Murray V, West C et al. (2013) 'Overheating and hospitals – what do we know?' J. Hosp.Adm. 2:1

4. HM Government (2020) *National Risk Register of Civil Emergencies*: https://assets.publishing.service.gov.uk/government/uploads/system/uploads/attachment_data/file/952959/6.6920_CO_CCS_s_National_Risk_Register_2020_11-1-21-FINAL.pdf

5. Climate Change Committee (2021) *Independent Assessment of UK Climate Risk. Advice to Government for the UK's Third Climate Change Risk Assessment:* https://www.theccc.org.uk/publication/independent-assessment-of-uk-climate-risk/

6. Met Office (2020) UK Monthly Climate Summary – Annual 2019: https://www.metoffice.gov.uk/binaries/content/assets/metofficegovuk/pdf/weather/learn-about/uk-past-events/summaries/uk_monthly_climate_summary_annual_2019.pdf

7. DHSC (2018) *The Heatwave Plan for England:* https://assets.publishing.service.gov.uk/government/uploads/system/uploads/attachment_data/file/888668/Heatwave_plan_for_England_2020.pdf

8. Short, A (2017) *The Recovery of Natural Environments in Architecture: Air, Comfort and Climate.* London: Taylor and Francis. In: Climate Change Committee (2014) *Climate Change Adaptation Committee Progress Report 2014 – Managing climate risks to wellbeing and the economy:* www.theccc.org.uk/wp-content/uploads/2014/07/Final_ASC-2014_web-version.pdf

For peer review only - http://bmjopen.bmj.com/site/about/guidelines.xhtml

9. Fifield, L. J., Lomas, K. J., Giridharan, R., & Allinson, D. (2018). Hospital wards and modular construction: Summertime overheating and energy efficiency. *Building and Environment*, 141, 28-44.

10. Climate Change Committee (2014) Climate Change Adaptation Committee Progress Report 2014 – Managing climate risks to wellbeing and the economy: www.theccc.org.uk/wp-content/uploads/2014/07/Final_ASC-2014_web-version.pdf

11. Health Technical Memorandum 03- 01: Specialised ventilation for healthcare premises- Part A: Design and validation: https://www.england.nhs.uk/publication/specialised-ventilation-for-healthcare-buildings/

12. NHS (2020) Estates Returns Information Collection Summary page and dataset for ERIC 2019/20: https://digital.nhs.uk/data-andinformation/publications/statistical/estates-returns-information-collection/england-2019-20

13. Casanueva A, Kotlarski S, Fischer A, Flouris A, Kjellstrom T, Nybo L, Schwierz C, Liniger M (2020) 'Escalating environmental summer heat exposure – a future threat for the European workforce.' *Regional Environmental Change,* 20:40

14. Climate and Health Alliance (2021) *Real, Urgent & Now: Insights from health professionals on climate and health in Australia.* Melbourne, Australia: Climate and Health Alliance

15. Flouris A, Petros D, Leonidas I, Lars N, Havenith G, Kenny G, Kjellstrom T (2018) 'Workers' health and productivity under occupational heat strain: a systematic review and meta-analysis.' *Lancet Planet Health,* 2:e521-31

16. Loosemore M and Chand A (2016) 'Barriers to Building Resilience to Extreme Weather Events in Australian Hospitals.' In: P W Chan and C J Neilson (Eds.) *Proceedings of the*

For peer review only - http://bmjopen.bmj.com/site/about/guidelines.xhtml

32nd Annual ARCOM Conference, 5-7 September 2016, Manchester, UK, Association of Researchers in Construction Management, 2: 1201-1210.

17. Carmichael C, Bickler G, Kovats S, Pencheon D, Murray V, West C, Doyle Y (2013) 'Overheating and hospitals – what do we know?' *Journal of Hospital Administration* 2:1

18. Gough, H., Faulknall-Mills, S., King, M.-F., & Luo, Z. (2019) 'Assessment of Overheating Risk in Gynaecology Scanning Rooms during Near-Heatwave Conditions: A Case Study of the Royal Berkshire Hospital in the UK.' *International Journal of Environmental Research and Public Health, 16*(18), 3347

19. Williams L, Erens B, Ettelt S, Hajat S, Manacorda T, Mays N (2019) *Evaluation of the Heatwave Plan for England – Final Report.* London: Policy Innovation and Evaluation Unit (PIRU)

20. Kovats S, and Brisley R, (2021) 'Health, communities and the built environment.' In: *The Third UK Climate Change Risk Assessment Technical Report* [Betts, R.A., Haward, A.B., Pearson, K.V. (eds.)]. Prepared for the Climate Change Committee: https://www.ukclimaterisk.org/independent-assessment-ccra3/technical-report/

For peer review only - http://bmjopen.bmj.com/site/about/guidelines.xhtml

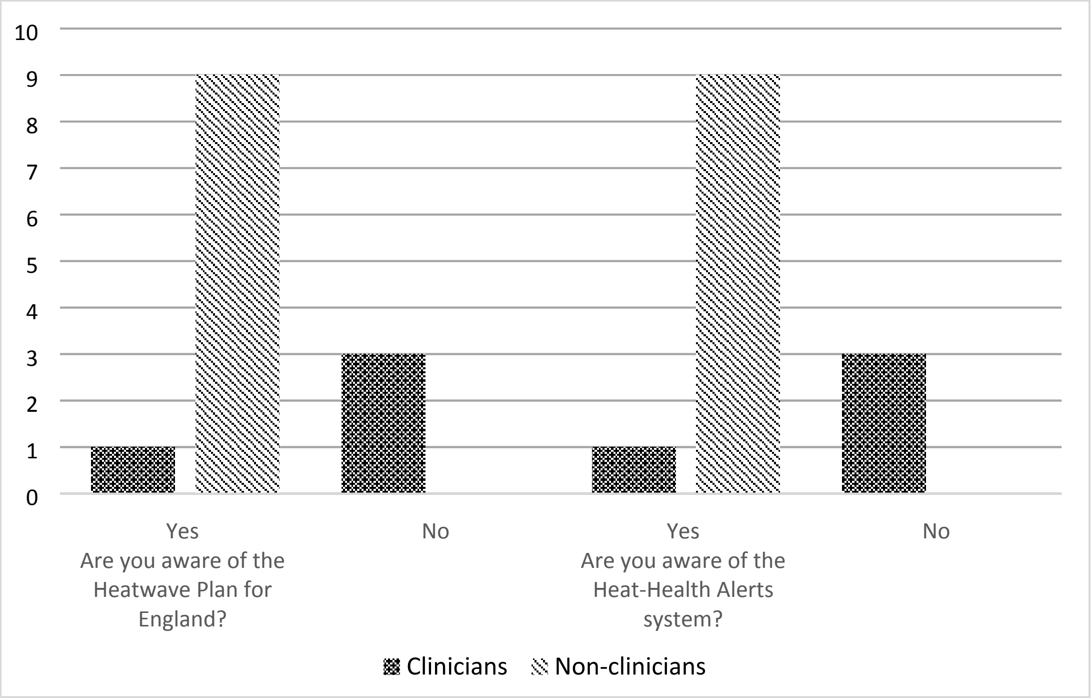

For peer review only

Standards for Reporting Qualitative Research (SRQR)*

http://www.equator-network.org/reporting-guidelines/srqr/

Title and abstract

	Page/line no(s).
**Title** - Concise description of the nature and topic of the study Identifying the study as qualitative or indicating the approach (e.g., ethnography, grounded theory) or data collection methods (e.g., interview, focus group) is recommended	Line 1
**Abstract** - Summary of key elements of the study using the abstract format of the intended publication; typically includes background, purpose, methods, results, and conclusions	Line 10

Introduction

**Problem formulation** - Description and significance of the problem/phenomenon studied; review of relevant theory and empirical work; problem statement	Line 54; line 63; Line 63
**Purpose or research question** - Purpose of the study and specific objectives or questions	

Methods

**Qualitative approach and research paradigm** - Qualitative approach (e.g., ethnography, grounded theory, case study, phenomenology, narrative research) and guiding theory if appropriate; identifying the research paradigm (e.g., postpositivist, constructivist/ interpretivist) is also recommended; rationale**	Line 69
**Researcher characteristics and reflexivity** - Researchers' characteristics that may influence the research, including personal attributes, qualifications/experience, relationship with participants, assumptions, and/or presuppositions; potential or actual interaction between researchers' characteristics and the research questions, approach, methods, results, and/or transferability	Line 84
**Context** - Setting/site and salient contextual factors; rationale**	Line 73
**Sampling strategy** - How and why research participants, documents, or events were selected; criteria for deciding when no further sampling was necessary (e.g., sampling saturation); rationale**	Line 74
**Ethical issues pertaining to human subjects** - Documentation of approval by an appropriate ethics review board and participant consent, or explanation for lack thereof; other confidentiality and data security issues	Line 70
**Data collection methods** - Types of data collected; details of data collection procedures including (as appropriate) start and stop dates of data collection and analysis, iterative process, triangulation of sources/methods, and modification of procedures in response to evolving study findings; rationale**	Line 82-90

For peer review only

**Data collection instruments and technologies** - Description of instruments (e.g., interview guides, questionnaires) and devices (e.g., audio recorders) used for data collection; if/how the instrument(s) changed over the course of the study	Line 82-90
**Units of study** - Number and relevant characteristics of participants, documents, or events included in the study; level of participation (could be reported in results)	Table 1
**Data processing** - Methods for processing data prior to and during analysis, including transcription, data entry, data management and security, verification of data integrity, data coding, and anonymization/de-identification of excerpts	Line 82-90
**Data analysis** - Process by which inferences, themes, etc., were identified and developed, including the researchers involved in data analysis; usually references a specific paradigm or approach; rationale**	Line 88-90
**Techniques to enhance trustworthiness** - Techniques to enhance trustworthiness and credibility of data analysis (e.g., member checking, audit trail, triangulation); rationale**	Line 87

Results/findings

**Synthesis and interpretation** - Main findings (e.g., interpretations, inferences, and themes); might include development of a theory or model, or integration with prior research or theory	Line 103
**Links to empirical data** - Evidence (e.g., quotes, field notes, text excerpts, photographs) to substantiate analytic findings	Eg Line 108

Discussion

**Integration with prior work, implications, transferability, and contribution(s) to the field** - Short summary of main findings; explanation of how findings and conclusions connect to, support, elaborate on, or challenge conclusions of earlier scholarship; discussion of scope of application/generalizability; identification of unique contribution(s) to scholarship in a discipline or field	Line 323; Line 341; Line 373; Line 361
**Limitations** - Trustworthiness and limitations of findings	

Other

**Conflicts of interest** - Potential sources of influence or perceived influence on study conduct and conclusions; how these were managed	None identified
**Funding** - Sources of funding and other support; role of funders in data collection, interpretation, and reporting	Line 393

*The authors created the SRQR by searching the literature to identify guidelines, reporting standards, and critical appraisal criteria for qualitative research; reviewing the reference lists of retrieved sources; and contacting experts to gain feedback. The SRQR aims to improve the transparency of all aspects of qualitative research by providing clear standards for reporting qualitative research.

For peer review only

For peer review only - http://bmjopen.bmj.com/site/about/guidelines.xhtml

**The rationale should briefly discuss the justification for choosing that theory, approach, method, or technique rather than other options available, the assumptions and limitations implicit in those choices, and how those choices influence study conclusions and transferability. As appropriate, the rationale for several items might be discussed together.

Reference:

O'Brien BC, Harris IB, Beckman TJ, Reed DA, Cook DA. **Standards for reporting qualitative research: a synthesis of recommendations.** *Academic Medicine*, Vol. 89, No. 9 / Sept 2014 DOI: 10.1097/ACM.0000000000000388

For peer review only

BMJ Open

Heatwaves, hospitals, and health system resilience in England: a qualitative assessment of frontline perspectives from the hot summer of 2019

Journal:	*BMJ Open*
Manuscript ID	bmjopen-2022-068298.R1
Article Type:	Original research
Date Submitted by the Author:	04-Jan-2023
Complete List of Authors:	Brooks, Katya; UK Health Security Agency (UKHSA), Extreme Events and Health Protection team<br>Landeg, Owen; UK Health Security Agency (UKHSA), Extreme Events and Health Protection team<br>Kovats, Sari; London School of Hygiene & Tropical Medicine, Dept of Social and Environmental Health Research<br>Sewell, Mark; NHS Improvement, National Emergency Preparedness Resilience and Response<br>OConnell, Emer; UK Health Security Agency, Extreme Events and Health Protection team
**Primary Subject Heading**:	Public health
Secondary Subject Heading:	Health services research, Health policy
Keywords:	PUBLIC HEALTH, Health & safety < HEALTH SERVICES ADMINISTRATION & MANAGEMENT, Quality in health care < HEALTH SERVICES ADMINISTRATION & MANAGEMENT, OCCUPATIONAL & INDUSTRIAL MEDICINE, QUALITATIVE RESEARCH, Health policy < HEALTH SERVICES ADMINISTRATION & MANAGEMENT

SCHOLARONE™
Manuscripts

I, the Submitting Author has the right to grant and does grant on behalf of all authors of the Work (as defined in the below author licence), an exclusive licence and/or a non-exclusive licence for contributions from authors who are: i) UK Crown employees; ii) where BMJ has agreed a CC-BY licence shall apply, and/or iii) in accordance with the terms applicable for US Federal Government officers or employees acting as part of their official duties; on a worldwide, perpetual, irrevocable, royalty-free basis to BMJ Publishing Group Ltd ("BMJ") its licensees and where the relevant Journal is co-owned by BMJ to the co-owners of the Journal, to publish the Work in this journal and any other BMJ products and to exploit all rights, as set out in our licence.

The Submitting Author accepts and understands that any supply made under these terms is made by BMJ to the Submitting Author unless you are acting as an employee on behalf of your employer or a postgraduate student of an affiliated institution which is paying any applicable article publishing charge ("APC") for Open Access articles. Where the Submitting Author wishes to make the Work available on an Open Access basis (and intends to pay the relevant APC), the terms of reuse of such Open Access shall be governed by a Creative Commons licence – details of these licences and which Creative Commons licence will apply to this Work are set out in our licence referred to above.

Other than as permitted in any relevant BMJ Author's Self Archiving Policies, I confirm this Work has not been accepted for publication elsewhere, is not being considered for publication elsewhere and does not duplicate material already published. I confirm all authors consent to publication of this Work and authorise the granting of this licence.

For peer review only

Title: Heatwaves, hospitals, and health system resilience in England: a qualitative assessment of frontline perspectives from the hot summer of 2019

Authors: Katya Brooks[1], Owen Landeg[1], Sari Kovats[2], Mark Sewell[3], Emer OConnell[1]

Corresponding author: EO (emer.publichealth@gmail.com)

[1]Extreme Events and Health Protection, UK Health Security Agency (UKHSA)

[2]NIHR Health Protection Research Unit in Environmental Change and Health (HPRU ECH), Faculty of Public Health and Policy, London School of Hygiene and Tropical Medicine

[3]National Emergency Preparedness Resilience and Response, National Health Service (NHS) England and NHS Improvement

Abstract: [228 words]

Objective: To critically assess the impacts of very hot weather on i) frontline staff in hospitals in England; and ii) on healthcare delivery and patient safety.

Study design: A qualitative study design using key informant semi-structured interviews, pre-interview survey and thematic analysis.

Setting: England

Participants: 14 health professionals in the National Health Service (NHS) (clinicians, non-clinicians including facilities managers and emergency preparedness, resilience and response (EPRR) professionals

Results: Hot weather in 2019 caused significant disruption to health services, facilities and equipment, staff and patient discomfort, and an acute increase in hospital admissions. Levels of awareness varied between clinical and non-clinical staff of the Heatwave Plan for England, Heat-Health Alerts and associated guidance. Response to heatwaves was affected

For peer review only - http://bmjopen.bmj.com/site/about/guidelines.xhtml

by competing priorities and tensions including infection control, electric fan usage and patient safety).

Conclusions: Healthcare delivery staff experience difficulty in managing heat risks in hospitals. Priority should be given to workforce development and strategic, long-term planning, prevention and investment to enable staff to prepare and respond, as well as to improve health system resilience to current and future heat-health risks. Further research with a wider, larger cohort is required to develop the evidence base on the impacts, including the costs of those impacts, and to assess the effectiveness and feasibility of interventions. Forming a national picture of health system resilience to heatwaves will support national adaptation planning for health, in addition to informing strategic prevention and effective emergency response.

Key words: hot weather, high temperatures, climate change, adaptation, public health, emergency planning, patient safety

Strengths and limitations

- This study explored the experiences of NHS employees during hot weather in geographically dispersed healthcare facilities across England in summer 2019.
- Participants interviewed included both clinical (n=4) and non-clinical (n=10) staff and provided a range of perspectives on how heatwaves affect health care delivery.
- The clinicians interviewed represented a limited range of clinical specialisms and future studies should look at how heatwaves affect a wide range of clinical care types.

For peer review only - http://bmjopen.bmj.com/site/about/guidelines.xhtml

- Interviews were conducted after a heatwave (England experienced three Level 3 Heat-Health Alerts in 2019).
- Interviews conducted via telephone or Skype may have limited participants' ability to communicate their experiences and interviewers' ability to interpret them.

1. Introduction

The frequency, intensity and duration of heatwaves is increasing in the United Kingdom (UK) due to anthropogenic climate change.(1-3) The relationship between human health and temperature is well established; heatwaves are already a public health concern and are considered a risk to national security.(4-8) Evidence from the UK's Third Climate Change Risk Assessment (CCRA3) suggests the heatwaves remain an under-managed risk, and will impact population health and health system delivery. Thus, heat is a priority risk for urgent action for England.(9) Healthcare infrastructure in England is generally not designed to cope with extreme heat and air conditioning is not routinely installed.(10) An estimated 90% of hospital buildings are vulnerable to overheating (11) and NHS estates are at risk of high indoor temperatures (overheating) even during moderately warm summers (12); temperatures in some wards can exceed 30˚C even when external temperatures are 22˚C.(13) Existing standards for healthcare premises recommend temperatures from 18˚C to 28˚C in general wards and 18˚C to 25˚C for more sensitive areas, such as birthing and recovery rooms.(12) In 2019-20, there were 3,600 instances of overheating above 26°C reported in NHS Trust buildings in England.(14) In addition to concerns about patient safety, heat is an occupational health hazard and can cause discomfort and harm to staff as well as affect productivity.(14-16)

For peer review only - http://bmjopen.bmj.com/site/about/guidelines.xhtml

Summer 2019 was warmer than average; with the highest daily temperature recorded in England (38.7°C in Cambridge) until temperatures were broken in the extremely hot summer of 2022.(17) The 2019 heatwave across Europe was made worse by anthropogenic climate change: temperatures were 1.5–3°C hotter than they would have been without human influence.(18)

A total of 15 Level 2 or Level 3 Heat-Health Alerts were issued across all regions of England in June, July and August 2019 based on the thresholds for the Heatwave Plan for England.(19) Public Health England (PHE) estimate that the excess mortality caused by the three heatwaves (level 3 alerts) in 2019 was approximately 892 deaths, the majority of which were in older persons.(20) However, there are no published studies of impacts on hospital admissions. PHE syndromic surveillance shows increases in emergency attendances for heat/sun stroke in line with these periods of hot weather.(21) The Heatwave Plan (19) aims to raise awareness of the health consequences of hot weather and describes what healthcare professionals can do to prepare and plan for hot weather along with actions to take in response to a heatwave. Actions for frontline staff in primary and secondary care include: identify and check on high-risk individuals and raise awareness of heat illnesses and their prevention among clients and carers; identify or create cool rooms/areas where temperatures can be maintained below 26°C and regularly monitor and record indoor temperatures and; ensure business continuity plans are in place and implemented as required.(19)

It has become a priority for the health system to better prepare for future heatwaves, as shown with the inclusion of severe weather response to the NHS Core Standards for Emergency Preparedness, Resilience and Response (EPRR).(22) However, there is limited

For peer review only - http://bmjopen.bmj.com/site/about/guidelines.xhtml

awareness that hot weather affects delivery of care and patient safety, and limited existing observational evidence about how healthcare settings manage episodes of hot weather. Anecdotal evidence for impacts has been reported during past heatwave events in England.(3, 23, 24) This paper addresses an important gap in the evidence by exploring the experiences of staff during hot weather in healthcare facilities in England in summer 2019 with a focus on the impacts on health service delivery, staff welfare, and patient safety.

2. Methods

Study design

A qualitative approach was conducted to collect in-depth data on the experiences of staff during hot weather in healthcare facilities in England in summer 2019 using a pre-interview survey and semi-structured interviews. Ethical approval was granted by PHE's Research Ethics and Governance Group in September 2019 (R&D reference: 366).

Sampling strategy

Participants were identified using snowball, purposive sampling to include those with a broad range of clinical and non-clinical expertise from healthcare facilities across England. Initial approaches were made to participants of the annual PHE heatwave seminar and colleagues identified by NHS England. Sample size was determined by applying the concept of saturation, with suggested individuals being invited and interviewed until no new themes or issues emerged, within the scope of available resources.

Recognising the differences in temperatures experienced across England, a geographical spread of participants from across the country was sought. It is important to emphasise that

DRAFT
For peer review only

the summer of 2019 was warm but not the extremes in temperature since seen in England. The study intentionally aimed to explore the experiences of the healthcare workforce dealing with overheating in healthcare estates as a routine summer issue, rather than their response to more unusual extreme heatwave conditions.

Data collection

Participants were invited by email to a one-hour telephone/Skype or face-to-face interview, depending on their geographical location, availability and preference. On acceptance, they were invited to complete a short pre-interview online questionnaire in SelectSurvey software, covering actions related to each Heat-Health Alert level (Level 2 and Level 3) from the Heatwave Plan to inform discussions during the interview. All participants provided written consent prior to participation.

Data collection instruments and technologies

Semi-structured interviews provided an open framework to engage in a guided discussion with participants on their experiences, challenges, learning and reflections of the period. An interview protocol and topic guide, based around a schedule of common questions, was piloted and employed to ensure consistency and enable comparison across interviews. Interviews comprised guided discussion on the following topics: background information; heatwave planning, preparing and alerting; impacts on participants, their colleagues and working environment; impacts on patients; and recommendations for building future heatwave resilience.

Units of study

For peer review only - http://bmjopen.bmj.com/site/about/guidelines.xhtml

134 Fourteen semi-structured interviews were completed by key informants in hospital settings comprising four clinical and 10 non-clinical employees of the NHS (including Estates and Facilities and Emergency Preparedness, Resilience and Response (EPRR) professionals) (Table 1) between October 2019 and January 2020.

Table 1. Breakdown of interviews by interview and role type

Code	Role type	Region
Non-clinical 1	Non-clinical (EPRR)	London
Non-clinical 2	Non-clinical (critical care)	London
Clinician 1	Clinician (acute medicine)	West Yorkshire
Non-clinical 3	Non-clinical (NHS E&I)	National / London
Non-clinical 4	Non-clinical (EPRR)	London
Clinician 2	Clinician (acute medicine)	Liverpool
Clinician 3	Clinician (junior doctor)	London
Non-clinical 5	Non-clinical (EPRR)	London
Non-clinical 6	Non-clinical (EPRR)	Berkshire
Non-clinical 7	Non-clinical (EPRR)	Kent
Non-clinical 8	Non-clinical (estates and facilities)	London
Clinician 4	Clinician	Manchester

For peer review only - http://bmjopen.bmj.com/site/about/guidelines.xhtml

Code	Role type	Region
Non-clinical 9	Non-clinical (EPRR)	London
Non-clinical 10 and Non-clinical 11 (Interviewed together)	Non-clinical (EPRR)	Kent

<u>Data processing</u>

For accuracy and transparency, interviews were recorded and transcribed verbatim, and all participants given the opportunity to review their interview transcripts. Each participant was assigned a participant number to ensure anonymity.

<u>Data analysis</u>

The dataset was thematically analysed and coded using EPPI-Reviewer (Evidence for Policy and Practice Information) software and a coding framework constructed through an iterative process that was both inductive and deductive, and trialled and agreed by the research team.

<u>Patient and public involvement</u>

The Health Protection Research Unit (HPRU) in Environmental Change and Health undertakes public involvement and engagement (PPI/E) for its research. Although the PPI/E group “Planet” was not established at the time this research was undertaken, there have been two workshops on heatwaves and health to plan further research and communicate results effectively to the general public.

3. Results

For peer review only - http://bmjopen.bmj.com/site/about/guidelines.xhtml

Thematic analysis identified the following key themes: impacts of heat on individual health and wellbeing (occupational health, and patient health and safety); impacts on healthcare provision; barriers to service delivery; heatwave planning; and consideration of adaptation to climate change.

Impacts of heat on staff and patients

The majority of participants reported experiencing overheating in healthcare buildings during the summer, with adverse effects on themselves and other staff and patients. Clinical and non-clinical respondents reported significant impacts of high temperatures on both staff and patients in terms of feeling uncomfortable, tired, stressed, unable to cope, less efficient, and observing distress in patients.

> *‘It had a huge impact on staff, but also on the patients as well, especially on the wards… some of them were acutely unwell at that time and exacerbated by very uncomfortable conditions’* (Non-clinical 14).

> *I remember… running around trying to get fans for the patients but the nurses…not having anything at the nurses' station, and people were generally just hot and stressed and looked uncomfortable. I remember one time we abandoned the handover because we just didn't cope in that room.* (Clinician 2)

Some facilities may be particularly prone to overheating due to factors in addition to building design. One EPRR Manager for a psychiatric unit reported that:

> *Especially for those patients… who are detained under the Mental Health Act and aren't allowed, or it's not safe for them to leave any of the wards…. some of the windows can't be opened for obvious reasons…. I know there was a few issues,*

For peer review only - http://bmjopen.bmj.com/site/about/guidelines.xhtml

understandably, family members were getting extremely distressed with staff saying, you know, this is unacceptable, [and I] totally understand it. … you have some kind of frustrations there amongst patients. We could see a rise in disruptive behaviour on the wards." (Non-clinical 9)

A high level of goodwill for maintaining patient care in the face of challenging working conditions on the wards was recognised by frontline clinicians: '*People did kind of pull together in adversity and everyone tried to keep the whole thing working'* (Clinician 3); *'That's probably the motto of the NHS: "just get on with it"'* (Clinician 4), as well as non-clinical staff: *'Our staff are really adaptable: whether it's hot or cold, they're really adaptable and the patients are at the heart of whatever they do and they check if our patients are ok in their own homes. Our staff are fantastic, they are brilliant'* (Non-clinical 10/11).

Increases in health service use, particularly for vulnerable adults

Respondents reported an increase in admissions on hot days: *'…you definitely notice that it's busier'* (Clinician 2). Patients were reported to present with physical symptoms of heat exhaustion, dehydration, cardiovascular and respiratory conditions, as well as '*injuries related to people doing kind of [outdoor] activities that you do in hot weather; so cycling, going on motorbike riding, drinking alcohol, things like that….'* (Clinician 4).

A specialist in acute medicine, reflected: *' [As well as] elderly people getting dehydrated, getting low blood pressure and having to come in because of fainting episodes and things like that and vulnerable groups that aren't able to rehydrate themselves, ask for water or reach the water because they've got dementia, or are very frail….'* (Clinician 4).

For peer review only - http://bmjopen.bmj.com/site/about/guidelines.xhtml

In addition to clinical risk factors, clinical respondents reported patients' housing or socio-economic status as putting them at greater risk in hot weather: *'[With one patient I remember] it was like late evening, so you can't speak to the GP and I remember kind of thinking, "My god, we're going to send him back to his [hot] flat which sounds like it's really precipitating continuous seizures and I don't know what kind of longer-term effects that's having…."'* (Clinician 3).

There were other concerns about discharging patients reported. Only two respondents, both EPRR Managers, reported protective procedures in place:

> *…[for] patients who are being discharged home during a Level 3 heatwave… we have a discharge proforma that gets filled in…. It highlights…these people will be at risk if they're on their own in a top floor flat with drugs that inhibit sweating, or inability to be walking around and getting drinks and keeping cool, for example. And then we would give those people a copy of the PHE [Beat the Heat] leaflet. Or is it safe to actually get them at home with some kind of social services input?* (Non-clinical 7)

Social isolation was also raised as a compounding factor: '*… people who were more socially isolated, perhaps living on their own, just managing most of the time, then weren't managing'* (Clinician 1).

Impacts of heat on facilities and equipment

Significant impacts on medical equipment and facilities that led to impacts on healthcare provision were reported. In relation to medical equipment, an EPRR Manager experienced failure of medication storage facilities in a psychiatric unit: *'Some of our medication, especially the anti-psychotic medication, needs to be kept at a specific temperature…. I*

For peer review only - http://bmjopen.bmj.com/site/about/guidelines.xhtml

believe on a couple of occasions the actual fridge which was containing some medication had bizarrely overheated, or just broke down' (Non-clinical 9). Two respondents reported issues due to server rooms overheating and as a result: *'they lost quite a lot of IT aspects, including the ability for all their different systems to talk to each other'* (Non-clinical 3).

An Estates and Facilities Manager reported high indoor temperatures in critical care units, operating theatres and rooms with MRI scanners which caused disruption to patient care, from loss of beds (which became unusable) and loss of equipment (scanners became unusable).

> *The intensive care unit was probably our worst area, and we struggled with some of our [MRI] scanners to keep them cool in certain periods as well, and that would have impacted on patient care. The paediatric scanning was suspended for periods; it was too unpleasant for the young ones to go through it.* (Non-clinical 8)

This view was echoed by an EPRR Manager from the same hospital:

> *Probably because the heat was rising and it just put enormous pressure on [our air conditioning unit] but the contingency didn't work, which meant that ... half of the ITU beds were lost or they were overheating…. We had to look at evacuating the critical care area because it was just getting too hot…. We moved some vulnerable out of those areas…. There were theatre suites that ... because the air conditioning was working so hard, they got full condensation so there was literally water running down on the walls and they had to stop operating… just to dry the walls, then deep clean it and then continue with the theatre list.* (Non-clinical 5)

For peer review only - http://bmjopen.bmj.com/site/about/guidelines.xhtml

Critical care facilities have a large number of air conditioners. These two respondents also reported that the increased demand during the hot weather caused a power failure which exacerbated indoor temperatures due to lack of air conditioning and also caused problems for refrigeration services as well as the mortuary: *'…. the mortuary fridges failed. In response, the Estates and Facilities Team 'converted a container for a temporary mortuary'* (Non-clinical 8). As a direct consequence of these concurrent events and their cumulative impacts on service delivery and patient safety, this hospital declared an internal incident due to the heatwave.

Barriers to service delivery in a heatwave

Several barriers to delivery of care in a heatwave were identified by respondents. These have been categorised in terms of preparedness and response to Heat-Health Alerts, built environment and managing indoor temperatures, and behaviours.

<u>Preparedness and response to Heat-Health Alerts</u>

Lack of time and capacity to respond to high temperatures due to competing priorities for frontline staff were reported across clinical and non-clinical respondents:

> *…Often when these things happen you're just so, so busy and it sometimes feels like you go from one crisis to another, that you don't actually have an awful lot of time to step back and say: "Right. What can we do differently?"* (Clinician 2)

Data from pre-interview questionnaires indicate a greater lack of awareness of the Heatwave Plan for England and Trust-level plans and Heat-Health Alerts amongst clinical respondents as compared with non-clinical respondents (see Figure 1).

For peer review only - http://bmjopen.bmj.com/site/about/guidelines.xhtml

Figure 1. Pre-interview questionnaire responses: awareness of the Heatwave Plan for England and the Heat-Health Alerts system

INSERT FIGURE

The majority of respondents reported that they received or cascaded Heat-Health Alerts by email. However, no clinician could recall receiving the alerts: ‘*Realistically as a [Junior Doctor] you’re probably not logging on to your emails, your NHS emails, that often*’ (Clinician 3). A reality recognised by some EPRR practitioners: ‘*[Email is] the best way to communicate really on heatwave alerts…. But, will they look at it? No way, not really*’ (Non-clinical 5) and ‘*It's a kind of, they’ll see on the news or something*’ (Non-clinical 9).

Across clinical respondents, a significant difference was observed in levels of awareness of the heat-health risks in general, in comparison with winter and cold weather:

> *Hot weather is never something that I've heard mentioned. Hot weather is almost one of those things you just get on and deal with….. Whereas all the time, even in the middle of summer, we're going to a wrap-up last winter meeting and then you go to a, what are we going to do next winter meeting.* (Clinician 2)

The majority of EPRR professionals interviewed identified the need for strategic, long-term planning, preparation and investment across the system to ensure healthcare resilience to all extreme weather hazards: ‘*The key thing for me, is about relationship building with [Estates and Facilities], so we will work closely with them all year round, not just when we suddenly get a heatwave alert*’ (Non-clinical 7).

For peer review only - http://bmjopen.bmj.com/site/about/guidelines.xhtml

> *We need to be much more prepared…. it's kind of frustrating really that we kind of deal with it when we're in the midst of it, you know…. So I think the lessons that we've learnt is almost we need to prepare from the turn of the year.* (Non-clinical 9)

> *Sometimes it's a challenge to get people to start thinking long term, and investment, money will always come down to being part of the issue….* (Non-clinical 1)

> *[The Heatwave Plan] gets circulated [annually] before our heat season starts and for people to say, please remember this. But the reality is that the majority of people have never opened it up and they'll kind of get almost kind of hit by it on the day when it gets hot. It's unfortunate it's a very reactive plan, rather than concentrating on this year-round.* (Non-clinical 5)

<u>Challenges of managing indoor temperatures</u>

Challenges of managing indoor temperatures often arise from the design of NHS buildings, which vary in the insulation and ventilation characteristics. Indoor temperatures are also increased by indoor heat sources (computers and equipment). Adaptation measures are limited by a lack of financial investment in the management of healthcare estates. One EPRR Manager reflected: *'The NHS is ageing and there's very little financially that we can do about it….'* (Non-clinical 5). A financial barrier reported by three non-clinical respondents stems from outsourced ownership of healthcare estate buildings, property management and maintenance leading to lack of control in making decisions to manage internal temperatures:

> *The majority of buildings are not owned by us…and we are tenants, and that's where we tend to get problems: There's normally quite a lot that's out of our control as it's*

DRAFT
For peer review only

For peer review only - http://bmjopen.bmj.com/site/about/guidelines.xhtml

> *a property services building so we can't necessarily do what we'd ideally want to do…. It would be nice to take control of own destinies.* (Non-clinical 10/11)

There is some evidence that new buildings are more adapted to hot weather. An EPRR Manager responsible for two sites commented that: *… Our [new] site, which is designed to kind of withstand heatwaves, we didn't have to activate any contingencies there at all… whereas at [the older site]… we definitely see that increase in [internal] temperature here…. we were getting… multiple incident forms every day in the summer.* (Non-clinical 7)

Infection prevention and control (IPC) policy was identified as a significant barrier to the use of electric fans, and to a lesser extent, air conditioning units in ward settings by all clinical and the majority of non-clinical respondents: '*Infection control is one of the overriding mantras everywhere'* (Clinician 1). Six respondents reported complete bans on electric fan usage in clinical areas '*We were just told that due to infection control we weren't allowed to use [electric fans] for patients'* (Clinician 4); '*They wouldn't allow fans because they said it was a health and safety risk'* (Clinician 3); '*…a clinical alert came out regarding…the fans…and said that… they're not to be used in clinical areas…. Because of infection control…and that came out just before the heatwave, you know, which made it quite difficult*' (Non-clinical 9). Others reported fans being used as expected, with inpatient areas prioritised: '*I remember people…running around trying to get fans for the patients and the nurses sort of not having anything at the nurses' station*' (Clinician 2). The difficulty of ensuring enough fans were available to provide sufficient cooling during heatwaves was cited: '*We had fans…. So all that was kind of in place, there just wasn't enough of it…. It's impossible to test like how many fans does it take to cool down this ward when it gets to 34 degrees*' (Non-clinical 5).

DRAFT
For peer review only

For peer review only - http://bmjopen.bmj.com/site/about/guidelines.xhtml

Two EPRR Managers stated that, due to financial and IPC constraints, the decision was made in their hospitals to annually dispose of electric fans and buy replacements to benefit patient care, if not the environment: ‘*Is it better to cool down the infectious patients with the fan, you know, and then chuck it, the fan, afterwards if you can't clean it, or let the poor man or woman just melt?’* (Non-clinical 5) and, *‘This is not great for the environment but it’s cheaper actually just to bin them and buy new fans’* (Non-clinical 1).

Most EPRR respondents reported annual procurement of portable air conditioning units: ‘*Where it's possible we bring in mobile air con units, but that's obviously not an ideal response’* <u>(Non-clinical 4).</u>

> *We can't purchase something portable and keep them in a cupboard, it's not a cost-effective way to do this. We will have to use an external company who can store them; you know, maintain them and test them and will bring them on site and hire them for that period of time.* (Non-clinical 5)

Two respondents reported instances where air conditioning could not be used across their sites: ‘*A lot of our corporate settings had the portable air conditioning units that could be used, but of course they can't be used in clinical settings because of infection control*’ (Non-clinical 9).

> *There were five…inpatient areas in old buildings that didn't have air conditioning installed, couldn’t - either for the size or the location of the ward, or the complexity of the patients’ [needs]…. We had outpatient areas where there could be children; you can't put any kind of air conditioning unit because of the risk of kiddies touching them. The only place with air conditioning in some areas would be in the middle of*

DRAFT
For peer review only

For peer review only - http://bmjopen.bmj.com/site/about/guidelines.xhtml

the nurses' station because of the risk of trip hazards, of the wires and of course, to elderly patients. (Non-clinical 5)

Participants called for increased clarity of infection control guidance at a national level as '*different trusts' infection control people say different things'* (Non-clinical 7) in order '*to demystify everything. For example, that air conditioning can or can't be used due to legionella'* (Non-clinical 6).

Participants questioned the feasibility of actions outlined in the Heatwave Plan, to create cool rooms and for checking indoor temperatures.

…Every bed is almost always full and there isn't necessarily extra space in the hospital to have a cooler area or the infrastructure to cool an area down…. it would be very challenging to implement actually, and I think… it would be difficult to kind of move someone out of the cool area into the hotter area in exchange for another patient, because you're subjecting them to a bit more discomfort and possibly risk as well. (Clinician 4)

… What if your coolest room is not near your nursing station, but you're going to put your vulnerable patients in there? ... Is it right that we put a vulnerable patient further away just because it's a bit cooler than anywhere else? (Non-clinical 1)

<u>The role of adaptive behaviours</u>

Heat risks can be reduced by changes in behaviour, in both frontline staff and patients. All participants reported lack of knowledge as a major challenge to adopting cooling strategies in health facilities, such as closing curtains or blinds, moving patients, etc.: *'…There doesn't*

For peer review only - http://bmjopen.bmj.com/site/about/guidelines.xhtml

appear to be any plan on it, but I think it was down to… nurses thinking we need to keep this patient in the shade’ (Clinician 1).

Workplace culture and attitudes were raised in relation as a barrier to staff drinking water and keeping hydrated whilst working on the ward due to fear of appearing unprofessional. One EPRR Manager sought to address this problem: ‘*We made it very clear to staff that we wanted to see them carrying water around, we wanted to see them drinking on duty, just to dispel all this old nonsense about, “oh you shouldn't be seen drinking on duty, it's unprofessional”. Actually, we said: “It is professional, it's looking after yourself”’* (Non-clinical 7).

4. Discussion

Our findings provide insights regarding the range of challenges experienced by staff and patients during hot weather in settings with limited processes and interventions in place to manage these conditions. Previous studies of individual hospitals and wards have identified factors related to the built environment and management of indoor temperatures (for example,(3, 23)), our findings broaden this perspective and identify wider considerations such as increased service demand, discomfort of patients and staff, and rooms and equipment becoming unusable or failing, leading to disruptions in service. It also highlights the importance of staff behaviours, clinical practice and organisational processes during heat episodes and how these factors may affect staff and patient wellbeing as well as healthcare delivery.

Barriers to care delivery associated with preparedness and response were also identified: capacity restraints, difficulties in identification and prioritisation of risk mitigating actions, gaps in awareness of heat-health risks and understanding of actions to take on receipt of an

For peer review only - http://bmjopen.bmj.com/site/about/guidelines.xhtml

alert were all cited.(25) These findings align with those of the *Evaluation of the Heatwave Plan for England*'s 2019 national survey of nurses in hospital, community and care home settings which reported that “many frontline staff, including nurses surveyed, reported to be unaware of any local heatwave plans, and unfamiliar with the Heatwave Plan for England guidance…many reported taking few or none of the recommended Heatwave Plan actions during an alert.”(26)

This research provides important insights on operational barriers to action, particularly in relation to use of electric fans as a cooling strategy and IPC concerns. Implementation of individual cooling interventions should form part of a whole systems approach that considers a hierarchy of cooling interventions, with active cooling as a last resort. The approach to cooling for any indoor environment should take account of the effectiveness, cost, feasibility, ease of use and sustainability of the different options in that context – in some settings, a combination of options may work best. This study has highlighted that a lack of clarity or consistency of policy on the use of fans and/or cooling systems has resulted in heat mitigations not being used and/or costly work-arounds being put in place due to concerns around IPC. Heat is one of several concurrent risks in these setting and trade-offs may be inevitable, but decisions should be supported by good evidence and this is currently lacking. Further, the routine disposal and replacement of electric fans does not align with Net Zero or sustainability commitments.

Future iterations of the Heatwave Plan for England should consider the barriers identified in this study and consider the feasibility of preventative action within clinical settings. Improvements in awareness and engagement with the existing Heatwave Plan and associated Heat-Health Alerts are needed, particularly for frontline staff. Efforts on

For peer review only - http://bmjopen.bmj.com/site/about/guidelines.xhtml

workforce development should focus on empowering and supporting staff to understand and act upon Heat-Health Alerts and to embed adaptive behaviours in their daily practice. Additionally, overheating should be considered/addressed within the forthcoming update to the National Adaptation Programme and included in the broader adaptation elements of NHS Green Plans.

For the medium to longer term, the diversity of the challenges identified highlights the need for a comprehensive assessment of the resilience of healthcare estates in England to inform future policy priorities. Ensuring a climate resilient health system will require strategic, long-term planning and investment to address the drivers for overheating risk and the barriers to action both before and during a heatwave event. At a minimum, any investment to improve energy efficiency should also consider adaptation.

<u>Limitations</u>

This study has several limitations. Interviews were conducted several months after the heatwave event, which may have led to recall bias. It is acknowledged that interviewees were more likely to be those who were more aware and had experienced difficulties during summer 2019. However, this is appropriate due to the aim of the paper to explore the pathways which heat affects health service delivery; not to provide a complete overview of the total impact. Relatively low engagement from clinicians in comparison with non-clinical NHS representatives may introduce a bias in terms of perspective. Interviews conducted via telephone or Skype may have limited participants' ability to communicate, and interviewers' ability to interpret, their experiences. However, this enabled engagement with participants working in more geographically dispersed locations across England than would otherwise have been possible.

For peer review only - http://bmjopen.bmj.com/site/about/guidelines.xhtml

5. Conclusion

This study is the first to consider the broad range of challenges faced by staff and patients when experiencing hot weather in healthcare estates in England. Many national public health systems are developing methods, plans and tools to manage climate risks, but adaptation is still largely viewed as an emergency response activity. Further action is needed to improve the climate resilience of the health system, in particular to address overheating in care settings, whilst committing to reaching carbon net zero by 2040.(27) The qualitative evidence presented here of the experiences of healthcare staff in England demonstrates that there is an issue, and it is not well quantified. Policies that support a climate resilient healthcare system need to be based on good evidence on the extent and scale of the problem. Specifically, additional research is recommended to further develop the evidence base on the impacts, viability of interventions - particularly in relation to a healthcare setting where IPC must also be considered - and current costs associated with heat-related disruption to health service delivery. This should inform a broader assessment of the climate vulnerability of the health system, and future planning assumptions for strategic prevention and emergency response.

6. Acknowledgements

The authors wish to thank the stakeholders that participated in this research.

7. Author statement

Contributors: KB and OL contributed equally to this paper. KB and OL designed the study, collected and analysed the data, drafted and revised the paper. MS contributed to the

For peer review only
DRAFT

design and implementation of the study. SK contributed to the design of the study. EO contributed to the design of the study, critically reviewed and finalised the draft paper.

8. Funding statement

This work was supported by the National Institute for Health Research (NIHR) Health Protection Research Unit in Environmental Change (NIHR200909), a partnership between the UK Health Security Agency (UKHSA) and the London School of Hygiene & Tropical Medicine. The views expressed are those of the author(s) and not necessarily those of the NIHR, UKHSA or the Department of Health and Social Care.

Ethics Approval Statement

Ethical approval was granted by Public Health England's Research Ethics and Governance Group in September 2019 (R&D reference: 366)

Data Availability Statement

No data are available: The participants of this study did not give written consent for their data to be shared publicly, so due to the small number of participants and the potential for them to be identified on the basis of their responses, the supporting data are not available.

Conflict of Interests Statement

None identified

References

1. Change IPoC. Summary for Policymakers. . Cambridge, United Kingdom and New York, NY, USA: Cambridge University Press; 2021.
2. Mitchell D HC, Vardoulakis S, Huntingford C, Masato G, Guillod B et al. . Attributing human mortality during extreme heatwaves to anthropogenic climate change. Environ Res Lett. 2016;11(7):8.
3. Carmichael C BG, Kovats S, Pencheon D, Murray V, West C et al. Overheating and hospitals – what do we know? J HospAdm 2013;2(1).
4. Government H. National Risk Register of Civil Emergencies. 2020.

For peer review only
DRAFT

5. Patel L CK, Sorenson C, McEachin S, Nadeau K, Kakkad K, Kizer KW. Climate Change and Extreme Heat Events: How Health Systems Should Prepare. NEJM Catalyst Innovations in Care Delivery. 2022;3(7).

6. Arnell N KA, Freeman A, Rudd A, Lowe J. Changing climate risk in the UK: a multi-sectoral analysis using policy-relevant indicators. Climate Risk Management. 2021;31.

7. Bikomeye JC RC, Beyer KMM. Positive Externalities of Climate Change Mitigation and Adaptation for Human Health: A Review and Conceptual Framework for Public Health Research. Int J Environ Res Public Health. 2021;18(5).

8. Blom IM BJ, Quintana AV. The COP26 health commitments: A springboard towards environmentally sustainable and climate-resilient health care systems? The Journal of Climate Change and Health. 2022:2667-782.

9. Committee CC. Independent Assessment of UK Climate Risk. Advice to Government for the UK's Third Climate Change Risk Assessment.; 2021.

10. Gupta R BL, Gregg M. Overheating in care settings: magnitude, causes, preparedness and remedies. Building Research and Information. 2017;45(1-2):83-191.

11. Short A. The Recovery of Natural Environments in Architecture: Air, Comfort and Climate. London: Taylor and Francis; 2017.

12. Committee CC. Climate Change Adaptation Committee Progress Report 2014 – Managing climate risks to wellbeing and the economy. 2014.

13. Fifield LJ LK, Giridharan R, Allinson D. . Hospital wards and modular construction: Summertime overheating and energy efficiency. Building and Environment. 2018;141:28-44.

14. NHS. Estates Returns Information Collection Summary page and dataset for ERIC 2019/20. 2020.

15. Casanueva A KS, Fischer A, Flouris A, Kjellstrom T, Nybo L, Schwierz C, Liniger M Escalating environmental summer heat exposure – a future threat for the European workforce. Regional Environmental Change. 2020;20.

16. Alliance CaH. Real, Urgent & Now: Insights from health professionals on climate and health in Australia. Melbourne, Australia: Climate and Health Alliance; 2021.

17. Office M. UK Monthly Climate Summary – Annual 2019. 2020.

18. Vautard R vAM, Boucher O, Drouin A, Haustein Km Kreienkamp F, van Oldenborgh GJ, Otto FEL, Ribes A, Robin Y, Schneider M, Soubeyrouz JM, Stott P, Seneviratne SI, Vogel MM, Wehner M. Human contribution to the record-breaking June and July 2019 heatwaves in Western Europe. Environmental Research Letters. 2020;15(9).

19. DHSC. The Heatwave Plan for England. 2018.

20. PHE. PHE heatwave mortality monitoring: Summer 2019. London: Public Health England; 2019.

21. England PH. Syndromic surveillance: weekly summaries for 2019. 2019.

22. NHS Core Standards for Emergency Preparedness, Resilience and Response [Internet]. 2019. Available from: https://www.england.nhs.uk/publication/nhs-england-core-standards-for-eprr/.

23. Flouris A PD, Leonidas I, Lars N, Havenith G, Kenny G, Kjellstrom T Workers' health and productivity under occupational heat strain: a systematic review and meta-analysis. Lancet Planet Health. 2018;2:e521-31.

24. A LMaC. Barriers to Building Resilience to Extreme Weather Events in Australian Hospitals. In: Neilson PWCaCJ, editor. Proceedings of the 32nd Annual ARCOM Conference, 5-7 September 2016; Manchester, UK: Association of Researchers in Construction Management, ; 2016. p. 1201-10.

25. Roberts T SV, Brooks K, Thompson R, Petrokofsky C, OConnell E, Landeg O. Stakeholder perspecives on extreme hot and cold weather alerts in England and the proposed move towards an impact-based approach. Environmental Science and Policy. 2022;136:467-75.

26. Gough H F-MS, King MF, Luo Z Assessment of Overheating Risk in Gynaecology Scanning Rooms during Near-Heatwave Conditions: A Case Study of the Royal Berkshire Hospital in the UK. International Journal of Environmental Research and Public Health. 2019;16(18).

For peer review only - http://bmjopen.bmj.com/site/about/guidelines.xhtml

27. Williams L EB, Ettelt S, Hajat S, Manacorda T, Mays N. Evaluation of the Heatwave Plan for England – Final Report. London: Policy Innovation and Evaluation Unit (PIRU); 2019.

DRAFT

For peer review only

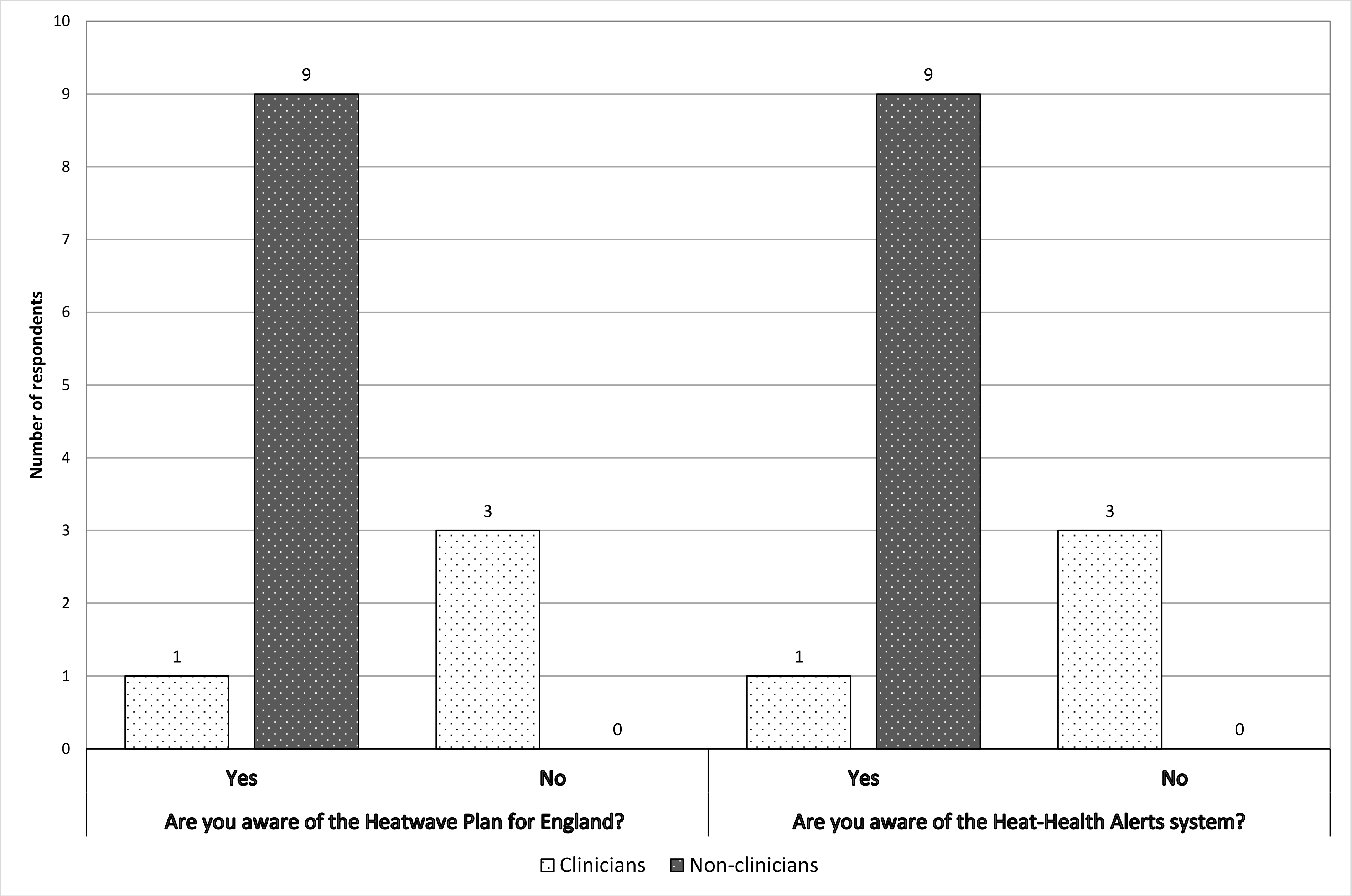

10
9
8
7
6
5
4
3
2
1
0
Number of respondents
9
9
3
3
1
1
0
0
Yes
No
Yes
No
Are you aware of the Heatwave Plan for England?
Are you aware of the Heat-Health Alerts system?
Clinicians
Non-clinicians

Standards for Reporting Qualitative Research (SRQR)*

http://www.equator-network.org/reporting-guidelines/srqr/

Title and abstract

	Page/line no(s).
**Title** - Concise description of the nature and topic of the study Identifying the study as qualitative or indicating the approach (e.g., ethnography, grounded theory) or data collection methods (e.g., interview, focus group) is recommended	Line 1
**Abstract** - Summary of key elements of the study using the abstract format of the intended publication; typically includes background, purpose, methods, results, and conclusions	Line 10

Introduction

**Problem formulation** - Description and significance of the problem/phenomenon studied; review of relevant theory and empirical work; problem statement	Lines 50-92
**Purpose or research question** - Purpose of the study and specific objectives or questions	Lines 92-95

Methods

**Qualitative approach and research paradigm** - Qualitative approach (e.g., ethnography, grounded theory, case study, phenomenology, narrative research) and guiding theory if appropriate; identifying the research paradigm (e.g., postpositivist, constructivist/ interpretivist) is also recommended; rationale**	Line 99-101
**Researcher characteristics and reflexivity** - Researchers' characteristics that may influence the research, including personal attributes, qualifications/experience, relationship with participants, assumptions, and/or presuppositions; potential or actual interaction between researchers' characteristics and the research questions, approach, methods, results, and/or transferability	Lines 101-102
**Context** - Setting/site and salient contextual factors; rationale**	Lines 100; 86-95
**Sampling strategy** - How and why research participants, documents, or events were selected; criteria for deciding when no further sampling was necessary (e.g., sampling saturation); rationale**	Lines 103-115
**Ethical issues pertaining to human subjects** - Documentation of approval by an appropriate ethics review board and participant consent, or explanation for lack thereof; other confidentiality and data security issues	Line 70
**Data collection methods** - Types of data collected; details of data collection procedures including (as appropriate) start and stop dates of data collection and analysis, iterative process, triangulation of sources/methods, and modification of procedures in response to evolving study findings; rationale**	Line 82-90

For peer review only
For peer review only - http://bmjopen.bmj.com/site/about/guidelines.xhtml

**Data collection instruments and technologies** - Description of instruments (e.g., interview guides, questionnaires) and devices (e.g., audio recorders) used for data collection; if/how the instrument(s) changed over the course of the study	Line 82-90
**Units of study** - Number and relevant characteristics of participants, documents, or events included in the study; level of participation (could be reported in results)	Table 1; lines 132-136
**Data processing** - Methods for processing data prior to and during analysis, including transcription, data entry, data management and security, verification of data integrity, data coding, and anonymization/de-identification of excerpts	Lines 138-141
**Data analysis** - Process by which inferences, themes, etc., were identified and developed, including the researchers involved in data analysis; usually references a specific paradigm or approach; rationale**	Linea 142-146
**Techniques to enhance trustworthiness** - Techniques to enhance trustworthiness and credibility of data analysis (e.g., member checking, audit trail, triangulation); rationale**	Line 145-146

Results/findings

**Synthesis and interpretation** - Main findings (e.g., interpretations, inferences, and themes); might include development of a theory or model, or integration with prior research or theory	Line 153-157; onwards
**Links to empirical data** - Evidence (e.g., quotes, field notes, text excerpts, photographs) to substantiate analytic findings	e.g. Line 164-170

Discussion

**Integration with prior work, implications, transferability, and contribution(s) to the field -** Short summary of main findings; explanation of how findings and conclusions connect to, support, elaborate on, or challenge conclusions of earlier scholarship; discussion of scope of application/generalizability; identification of unique contribution(s) to scholarship in a discipline or field	Lines 379-424
**Limitations** - Trustworthiness and limitations of findings	Lines 425-436

Other

**Conflicts of interest** - Potential sources of influence or perceived influence on study conduct and conclusions; how these were managed	None identified
**Funding** - Sources of funding and other support; role of funders in data collection, interpretation, and reporting	Lines 462-467

*The authors created the SRQR by searching the literature to identify guidelines, reporting standards, and critical appraisal criteria for qualitative research; reviewing the reference lists of retrieved sources; and contacting experts to gain feedback. The SRQR aims to improve the transparency of all aspects of qualitative research by providing clear standards for reporting qualitative research.

For peer review only - http://bmjopen.bmj.com/site/about/guidelines.xhtml

**The rationale should briefly discuss the justification for choosing that theory, approach, method, or technique rather than other options available, the assumptions and limitations implicit in those choices, and how those choices influence study conclusions and transferability. As appropriate, the rationale for several items might be discussed together.

Reference:

O'Brien BC, Harris IB, Beckman TJ, Reed DA, Cook DA. **Standards for reporting qualitative research: a synthesis of recommendations.** *Academic Medicine*, Vol. 89, No. 9 / Sept 2014 DOI: 10.1097/ACM.0000000000000388

For peer review only
